# Breaking diagnostic and therapeutic barriers in intravascular large B-cell lymphoma: A 13-year real-world study from China

**DOI:** 10.1186/s13023-026-04226-4

**Published:** 2026-02-11

**Authors:** Wei Wang, Danqing Zhao, Congwei Jia, Chong Wei, Yan Zhang, Wei Zhang, Daobin Zhou

**Affiliations:** 1 Department of Hematology, Peking Union Medical College Hospital, No.1 Shuaifuyuan, Dongcheng District, Beijing, 100005 China; 2 Department of Pathology, Peking Union Medical College Hospital, Beijing, China

**Keywords:** Intravascular large B-cell lymphoma, Ultra-orphan disease, Interleukin-10, Random skin biopsy, Zanubrutinib

## Abstract

**Background:**

Intravascular large B-cell lymphoma (IVLBCL) is an ultra-orphan disease (incidence 0.095/million/year) with dismal prognosis due to delayed diagnosis and limited therapeutic options. We aimed to evaluate a novel diagnostic algorithm and the efficacy of zanubrutinib-containing therapy in a real-world Chinese cohort.

**Methods:**

This single-center retrospective study enrolled 54 IVLBCL patients (2010–2022). Diagnostic approaches evolved from PET/CT-guided biopsy to a combination of serum interleukin-10 (IL-10, cut-off 95.65 pg/mL), random skin biopsy (RSB), and circulating tumor DNA (ctDNA) profiling. Treatment regimens shifted from R-CHOP to methotrexate-based therapy (MTX), then to zanubrutinib plus R-CHOP (ZR-CHOP).

**Results:**

The cohort exhibited distinct features: 35.2% Asian-variant IVLBCL, 50% CNS involvement, and 22.9% PET/CT negativity. IL-10 combined with RSB enabled the diagnosis of 11 PET/CT-negative patients who would have otherwise remained undiagnosed. ctDNA revealed *MYD88 L265P* (11/17, 65%) and *CD79B* (7/17, 41%) variants. ZR-CHOP (*n* = 22) achieved significantly superior 2-year progression-free survival (PFS) compared to R-CHOP alone (*n* = 6) (90% vs. 30%; hazard ratio [HR] 0.031, *P* < 0.0001) and demonstrated 100% central nervous system (CNS) relapse-free survival. The efficacy of ZR-CHOP was comparable to that of methotrexate (MTX)-based therapy (*n* = 20; 2-year PFS 85%), despite a shorter median follow-up (20.1 vs. 38.0 months).

**Conclusions:**

In this largest Asian IVLBCL cohort to date, IL-10 + RSB + ctDNA significantly improved diagnostic accuracy. Zanubrutinib demonstrated promising efficacy, particularly in CNS involvement, offering a pragmatic solution for this orphan disease.

**Supplementary Information:**

The online version contains supplementary material available at 10.1186/s13023-026-04226-4.

## Introduction

Intravascular large B-cell lymphoma (IVLBCL) is an extremely rare subgroup of diffuse large B‑cell lymphoma, with an incidence estimated at 0.095 per 1 million per year (SEER) [1]. IVLBCL belongs to extranodal lymphoma, which is characterized by diffuse and obliterative proliferation of lymphoma cells in tissues, organs, and lumens of small and medium vessels [2]. A peculiar characteristic of the disease is the lack of lymphadenopathy, and it develops with nonspecific symptoms such as fever, general fatigue, hypoxia, and unexplained neurological symptoms, which hamper timely and precise diagnosis. The existence of the disease is suspected only after the appearance of systemic symptoms mentioned above, and the delay in diagnosis directly leads to deteriorating general conditions due to progression [3].

In recent years, great effort has been made to make timely diagnosis of IVLBCL. Random skin biopsy(RSB) was initially mentioned in case reports in the early 2020s, which helped clinicians to make diagnosis of IVLBCL after weeks of exhaustive evaluations for fever of unknown origin (FUO) [4, 5]. After that, RSB has been gradually accepted as a useful tool for early diagnosis, with a sensitivity and specificity of 77.8% and 98.7%, respectively [6]. Besides, due to the increasing understanding of genetic profile of IVLBCL, liquid biopsy to detect mutations of *L265P MYD88* and *CD79B* was proposed as a useful diagnostic tool under certain circumstances when adequate pathological tissue was unavailable [7]. A case diagnosed by liquid biopsy has already been reported [8].

Advances in diagnostic approaches, specifically the IL-10/RSB algorithm, enabled the identification of more patients with IVLBCL, particularly those with negative PET/CT who were previously missed, allowing timely intervention and ultimately improving survival outcomes. Parallel therapeutic innovations have been equally crucial. The introduction of rituximab to CHOP (R-CHOP) revolutionized IVLBCL management, achieving 3-year overall survival (OS) rates of 81% in Western populations and 2-year progression-free survival (PFS)/OS of 56%/66% in Asian patients [9–11]. Subsequent recognition of frequent CNS involvement prompted CNS-directed strategies, with the PRIMEUR-IVL study demonstrating that adding high-dose methotrexate (3.5 g/m²) to R-CHOP improved 2-year PFS to 76% while reducing CNS relapse to just 3% [12].

Building upon these milestones, our center has refined IVLBCL management through diagnostic, monitoring, and treatment optimization [13]. This retrospective study evaluates how these evolving practices have influenced outcomes in this rare disease.

## Methods

### Overview

This retrospective single-center study included all IVLBCL patients treated at Peking Union Medical College Hospital (PUMCH) from January 2010 to December 2022. We extracted comprehensive demographic, clinical, radiologic, pathologic, and therapeutic data from medical records.

To evaluate temporal trends in management, we stratified the study period into three eras based on diagnostic and therapeutic advancements:

1. 2010–2016: Pre-IL-10 testing era (PET/CT-guided biopsies).
2. 2017–2019: IL-10 era (serum IL-10 testing implemented in late 2016).
3. 2020–2022: Multi-modal era (routine random skin biopsy [RSB], ctDNA analysis, and novel therapeutics introduction)

The evolution of our diagnostic strategy over the study period is visualized in Supplementary Figure S1A.

Notably, the stratification by calendar era is solely intended to demonstrate the evolution of our diagnostic and therapeutic strategies for IVLBCL. Survival analyses were stringently grouped by treatment regimen. Acknowledging that diagnostic capabilities and supportive care varied across eras, we mitigated these and other potential confounders by performing a multivariable analysis adjusted for key baseline parameters.

### Laboratory and pathological analyses

#### IL-10 assay

Serum IL-10 levels were measured using a an electrochemiluminescence immunoassay analyzer (Siemens Immulite 1000 and its corresponding IL-10 detection kit) according to the manufacturer’s instructions. The analytical performance and the diagnostic cut-off value of 95.65 pg/mL for IVLBCL in our institution were established in our previous validation study, with a diagnostic sensitivity of 80% and specificity of 100% [13], and the same protocol was applied to all patients in this cohort.

#### Random skin biopsy (RSB)

RSB was performed on clinically normal-appearing skin, typically from the thigh, upper arm, or abdomen. Under local anesthesia, at least two skin tissue specimens of 1 cubic centimeter each were obtained by surgeon, ensuring the sample included the full thickness of the dermis and subcutaneous fat. Specimens were fixed in formalin and embedded in paraffin. Sections were stained with hematoxylin and eosin (H&E) and subjected to immunohistochemical (IHC) analysis for CD20, CD3, and CD5 et al. The diagnosis of IVLBCL was established by the identification of atypical lymphoid cells staining positive for CD20 within the lumina of small vessels in the dermis and subcutis.

#### ctDNA sequencing and analysis

Plasma and matched peripheral blood leukocytes (PBLs) were collected as control. ctDNA was extracted from plasma using the QIAamp Circulating Nucleic Acid Kit (Qiagen), and germline DNA was extracted from PBLs using the DNeasy Blood & Tissue Kit (Qiagen). Sequencing libraries were constructed with the KAPA Hyper Library Prep Kit (KAPA Biosystems). Target capture was performed using the Geneseeq Prime™ panel (Nanjing Geneseeq Technology Inc.), which covers 425 genes known to be recurrently mutated in hematologic malignancies. The captured libraries were sequenced on an Illumina HiSeq4000 platform. The mean sequencing depth was > 5000x for cfDNA samples and 100x for matched PBL controls.

Circulating tumor DNA (ctDNA) analysis was performed using a panel that screened for single nucleotide variants (SNVs), insertions/deletions (INDELs), copy number variants (CNVs), and structural variants (SVs). All cases were analyzed for the presence of these alteration types. For a comprehensive list of all detected SNVs and the occurrence of CNA/SV events by gene, see Supplementary Table 4. It is noted that while CNV/SV events were called, their precise characterization was not available for all cases and they are reported at the general event level.

### Addressing potential confounding

To address potential confounding by calendar era and indication, we pre-specified three management eras and compared baseline characteristics (Table 3). Crucially, we performed multivariable Cox regression for PFS and OS, IPI score, and CNS involvement, which confirmed that the treatment effect of CNS-oriented therapy was independent of these factors.

### Statistical analysis

Continuous variables are presented as mean ± standard deviation (SD) or median (range or interquartile range [IQR]), as appropriate. Categorical variables are presented as frequencies and percentages (n, %). Specific statistical tests were applied as follows:

For comparisons of continuous variables: The unpaired t-test was used for normally distributed data between two groups; the Mann-Whitney U test was used for non-normally distributed data between two groups. For comparisons across more than two groups (e.g., the time from symptom onset to diagnosis across calendar eras), the Kruskal-Wallis test was employed.

For comparisons of categorical variables (e.g., complete remission [CR] rates): The Fisher’s exact test was used.

For survival analysis: Progression-free survival (PFS) and overall survival (OS) were estimated using the Kaplan-Meier method and compared with the log-rank test. To identify independent predictors of survival and to adjust for potential confounders (such as calendar era and CNS involvement), a multivariable Cox proportional hazards regression analysis was performed.

All statistical analyses were performed using SPSS (version 29.0; IBM Corp., Armonk, NY, USA), with the exception of Kaplan-Meier survival analysis and the generation of survival curves, which were conducted using GraphPad Prism 10.0 (GraphPad Software, San Diego, CA, USA) to leverage its specialized and high-quality graphical output.

## Results

### Patient characteristics (Table 1)

**Table 1:** Baseline characteristics of the entire cohort (*N* = 54)

	Overall
Number of patients	54
Age mean(± SD)	57.4(± 9.3)
Age ≥ 60 years n(%)	24(44.4%)
Sex = Female n (%)	24(44.4%)
Ann Arbor Stage IV	54(100%)
ECOG performance status	
0–2	22(40.7%)
3	13(24.1%)
4	19(35.2%)
International prognostic index risk group	
low risk	0(0%)
low-intermediate risk	2(3.7%)
intermediate-high risk	13(24.1%)
high risk	39(72.2%)
Presented with	
B symptom	49(90.7%)
Shock	10(18.5%)
Hypoxia	28(51.9%)
HLH	19(35.2%)
Variant	
Western type	35(64.8%)
Asian type	19(35.2%)
Isolated skin type	0(0%)
Involved site	
Lung	33(61.1%)
CNS	27(50.0%)
Skin	23(42.6%)
Bone marrow	23(42.6%)
Spleen	15(27.8%)
Bone	8(14.8%)
Pituitary gland	7(13.0%)
Kidney	6(11.1%)
Liver	6(11.1%)
Lymph node	5(9.3%)
Adrenal gland	4(7.4%)
Peripheral blood	4(7.4%)
GI tract	3(5.6%)
Muscle	3(5.6%)
Nasal cavity	2(3.7%)
Gall bladder	1(1.9%)
Heart	1(1.9%)
Median number of involved sites, n(range)	3(1–7)
Patient with > 1 extra nodal invovement, n(%)	48(88.9%)
Patient with unrevealing PET/CT	11/48(22.9%)
Cell of origin according to Han’s classifier	
non-GCB	35(64.8%)
GCB	5(9.3%)
Can not be classified	14(25.9%)
Double expresser	11/26 (42.3%)
CD5 possitive	14/31(45.2%)
EBER negative	13/13(100%)

Abrrevations: SD: standardized deviation, ECOG: Eastern Cooperative Oncology Group, HLH: hemophagocytic lymphohistiocytosis, CNS: central nervous system, GCG: germinal center B-cell-like, EBER: EBV-encoded RNA

#### Clinical features

Our study included 54 consecutive IVLBCL patients (2010–2022) with a mean age of 57.4 ± 9.3 years (male: female = 1.25:1). All patients presented with Ann Arbor stage IV disease, with 72.2% (39/54) classified as high-risk by IPI and 59.3% (32/54) having poor ECOG status (3–4). The Asian variant was identified in 35.2% (19/54) of cases. Key clinical features included: B symptoms (90.7%), hypoxia (51.9%), HLH (35.2%), shock (18.5%). Multiorgan involvement was common (median 3 sites, range 1–7), with predominant involvement of: lung (61.1%), CNS (50.0%), skin (42.6%), bone marrow (42.6%). Notably, 22.9% (11/48) of PET/CT scans were negative, requiring alternative imaging (CT/MRI) for diagnosis.(Details were presented in Table 1).

#### Pathological features (Figure S1B, Table 1)

Diagnostic biopsies were most frequently obtained from: skin (36.2%), bone marrow (15.5%),lung (15.5%). Immunophenotypic analysis revealed: non-GCB subtype: 87.5% (35/40), double-expressor lymphoma: 42.3% (11/26), CD5 positivity: 45.2% (14/31), All EBV-ISH negative cases (13/13)(Table 1).

#### Serum IL-10 and genomic alteration by circulating tumor DNA (Figure S2)

From May 2016 to December 2022, thirty-seven patients had baseline serum IL-10 tested. Median level of IL-10 was 331pg/ml (range: 5.0 to >1000pg/ml). 29(78.4%) patients had an IL-10 level more than 95.65 pg/mL, which was defined as the cut-off value with a diagnostic sensitivity of 80% and specificity of 100% for IVLBCL in our previous study [13].

From the year 2020, we began to apply ctDNA assay for patients who could afford it. 17 patients had genetic analysis. Results showed a high prevalence of *MYD88 L265P* (11/17, 65%) and *CD79b* (7/17, 41%) variants. Other genetic alteration was shown in Supplemental Tables 4 and FigureS2. Most of these changes enriches in pathway of B cell receptor, immune escape or *JAK/STAT* signaling.

### Evolution of diagnostic approaches (2010–2022) (Fig. 1; table 2, table S1)

**Fig. 1:**
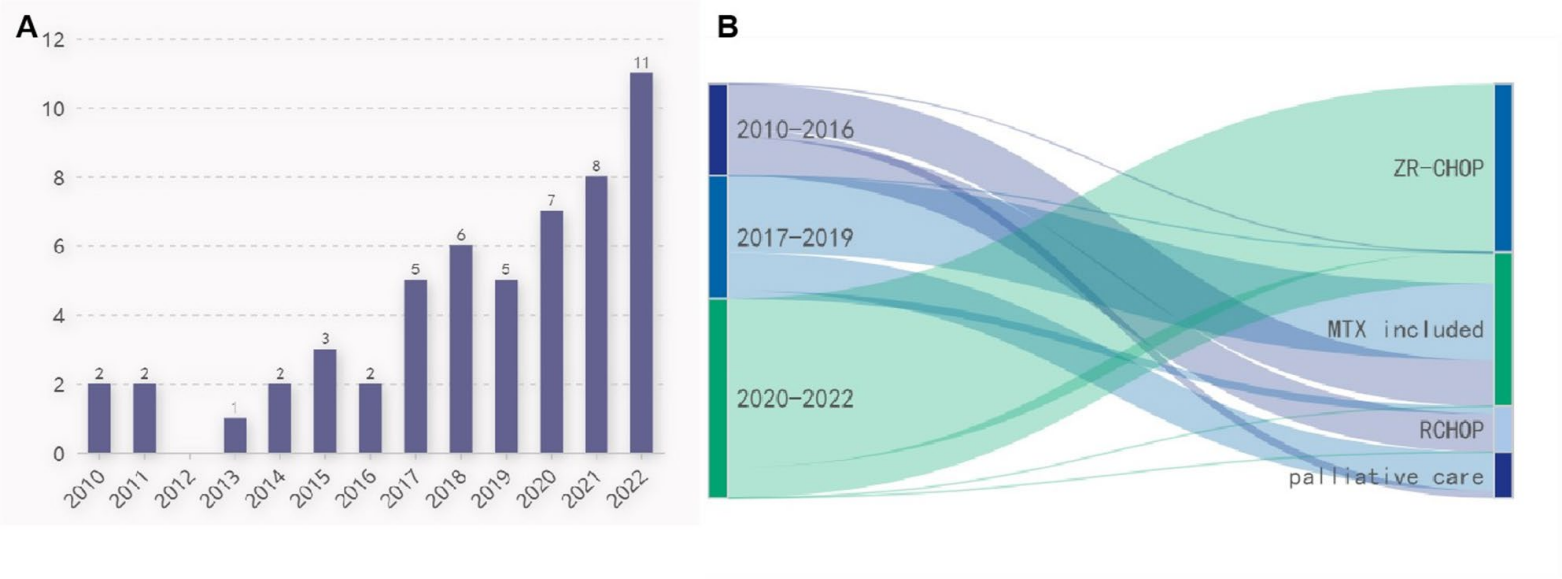
Evolution of clinical practice for IVLBCL. **A**. Number (on top of each column) of newly diagnosed IVLBCL patients each year. **B**. Sankey diagram of treatment in different time periods

**Table 2:** Comparing clinical approaches of patients in different time frames

Year	2010–2016	2017–2019	2020–2022	*P* value
Number of patients	12	16	26	NA
ctDNA tested	0(0%)	0(0%)	17(65.4%)	**< 0.001**
Plasm IL-10 tested	1(8.3%)	12(75%)	24(92.3%)	**< 0.001**
Ramdon skin biopsy upplied	1(8.3%)	10(62.5%)	23(88.5%)	**< 0.001**
Negative PET/CT	0(0%)	2/13(15.4%)	9/22(40.9%)	**0.022**
Diagnositic biopsy site in PET/CT negative patients				NA
Ramdon skin biopsy	NA	2(100%)	6(66.7%)	
Lung	NA	NA	1(11.1%)	
Spleen	NA	NA	1(11.1%)	
lymph node	NA	NA	1(11.1%)	
Time from Symptom Onset to Definitive Diagnosis Median(IQR)	3.1(1.9–3.9)	3.6(2.3–9.2)	4.8(2.2–6.4)	**0.197**
Treatment				**< 0.001**
ZR-CHOP	0(0%)	0(0%)	22(84.6%)	
MTX included therapy	6(50%)	10(62.5%)	4(15.4%)	
RCHOP only	5(41.7%)	1(6.3%)	0(0%)	
Palliative care	1(8.3%)	5(31.3%)	0(0%)	

Abrrevations: ECOG: Eastern Cooperative Oncology Group, CNS: central nervous system, IL-10: interleukin-10, IQR: interquartile range, MTX: methotrexate

Note: * difference in skin involvement should be interpreted as that widely application of random skin biopsy discovered more existing involvement of skin without any visible abnormalities

As mentioned before, we artificially divided the thirteen years in to three eras: 2010–2016, 2017–2019, and 2020–2022 since assessment for patients has been changing over these periods. In the early years (2010–2016), we diagnosed IVLBCL mainly by PET/CT guided biopsy, as we did to all other DLBCL patients. From the year of 2017, when we found that elevated serum IL-10(sIL-10) was a sensitive and specific marker for IVLBCL [13], we ordered this test for patients suspected of IVLBCL and applied random skin biopsy for those who had an elevated sIL-10 level. After 2020, combination of these two tests was widely applied and ctDNA examination was added to our clinical routine (Table 2). This diagnostic evolution was critical for identifying a substantial subset of patients with PET/CT-negative disease, a population that was previously largely undiagnosable. The impact of this shift is reflected in the changing proportion of PET/CT-negative cases over time: as detailed in Table 2, no such cases were identified in the 2010–2016 period when biopsies were predominantly PET/CT-guided. In contrast, the implementation of systematic IL-10 testing and random skin biopsy led to the identification of PET/CT-negative patients in 15.4% (2/13) and 40.9% (9/22) of cases in the 2017–2019 and 2020–2022 periods, respectively. Critically, this rising detection of PET/CT-negative cases was not accompanied by a reduction in the time from symptom onset to diagnosis across the eras (median 3.1 vs. 3.6 vs. 4.8 days, *P* = 0.197, see Table 2), effectively ruling out substantial lead-time bias as an explanation for the improved outcomes in later periods. This trend collectively underscores a fundamental change in case ascertainment rather than a true change in disease biology or a mere shift in diagnostic timing. The clinical features of the 11 PET/CT-negative patients are summarized in Table S1. This cohort, characterized by non-specific symptoms and poor performance status, exemplifies the patient population that would have eluded diagnosis without the sIL-10/RSB algorithm. The success of this proactive diagnostic approach is directly reflected in the rising annual number of new diagnoses, which peaked at 11 in 2022 (Fig. 1A).

### Therapeutic evolution across study periods (Fig. 1.B)

Besides the evaluation methods, treatment also changed dramatically during these years. In 2010–2016, RCHOP and RCHOP combined with intravenous methotrexate (MTX) was the mainstay of intervention. In our center, DLBCL patients with high risk of CNS relapse were given intravenous MTX at a dose of 1 g/m^2^ incorporated into RCHOP (the so-called R-MTX-CHOP regimen) for 4 courses, which yielded a good clinical outcome with low incidence of CNS progression [14]. For patients with CNS involvement at diagnosis, high dose MTX (3.5 g/m^2^) based therapy was given. R-MTX-CHOP and high dose MTX based therapy were called MTX included regimen in present study. During the year 2017–2019 when the importance of CNS prophylaxis was addressed, RCHOP regimen was phased out, patients were mainly treated by MTX included therapy. In 2020–2022, when realizing that most of the IVLBCL patients could be categorized into MCD subtype, we began to explore the efficacy of bruton kinase inhibitor, zanubrutinib, combined with RCHOP, the so-called ZR-CHOP regimen. Patients were treated by 8 cycles of RCHOP regimen combined with zanubrutinib 160 mg twice daily, and then zanubrutinib maintenance for two years. As a result, most of patients in this period were treated by ZR-CHOP, only four patients received MTX containing therapy. No patient chose palliative care in 2020–2022, while there were one(8.3%) and five(31.3%) patients receiving only support therapy in 2010–2016 and 2017–2019, respectively(Fig. 1.B).

### Survival outcomes by treatment modality (Table 3; Figs. 2 and 3,Table S2)

**Table 3:** Comparing clinical features of patients by different treatment

	ZR-CHOP	MTX included	RCHOP	Palliative care	*P* value
Number of patients	22	20	6	6	
Age, years median(range)	59.1 ± 7.8	56.9 ± 7.4	55.8 ± 15.7	54.0 ± 13.5	0.627
Age ≥ 60 years n(%)	11(50.0%)	8(40.0%)	2(33.3%)	4(50%)	0.848
Sex = Female n(%)	8(36.4%)	10(50.0%)	4(66.7%)	2(33.3%)	0.523
ECOG = 4 n(%)	9(40.9%)	5(25%)	1(16.7%)	4(66.7%)	0.206
IPI high risk n(%)	19(86.4%)	10(50%)	4(66.7%)	6(100%)	**0.022**
Shock n(%)	3(13.6%)	5(25%)	0(0%)	2(33.3%)	0.394
Hypoxia n(%)	12(54.4%)	7(35%)	4(66.7%)	5(83.3%)	0.154
HLH n(%)	9(40.9%)	6(30%)	2(33.3%)	2(33.3%)	0.943
Involved site n(%)					
Lung	15(68.2%)	9(45.0%)	4(66.7%)	6(100%)	0.085
CNS	14(63.6%)	8(40.0%)	2(33.0%)	3(50.0%)	0.452
Skin	14(63.6%)	6(30.0%)	1(16.7%)	2(33.3%)	0.073
Bone marrow	8(36.4%)	7(35.0%)	4(66.7%)	4(66.7%)	0.304
Anemia n(%)	15(68.2%)	13(65.0%)	5(83.3%)	5(83.3%)	0.835
Thrombocytopenia n(%)	11(50.0%)	13(65.0%)	3(50%)	3(50%)	0.774
LDH elevated n(%)	18(81.8%)	20(100.0%)	6(100.0%)	5(83.3%)	0.164
LDH > 1000U/L n(%)	10(45.5%)	9(45%)	3(50%)	1(16.7%)	0.699
CRR	90.9%(20/22)	90.0%(18/20)	66.7%(4/6)	NA	0.309^#^

Abrrevations: MTX: methotrexate, ECOG: Eastern Cooperative Oncology Group, IPI: international prognostic index, HLH: hemophagocytic lymphohistiocytosis, CNS: central nervous system, IL-10: interleukin-10, LDH: lactate dehydrogenase, CRR: complete response rate

#Patients who received palliative care were not included in the comparison of CRR

**Fig. 2:**
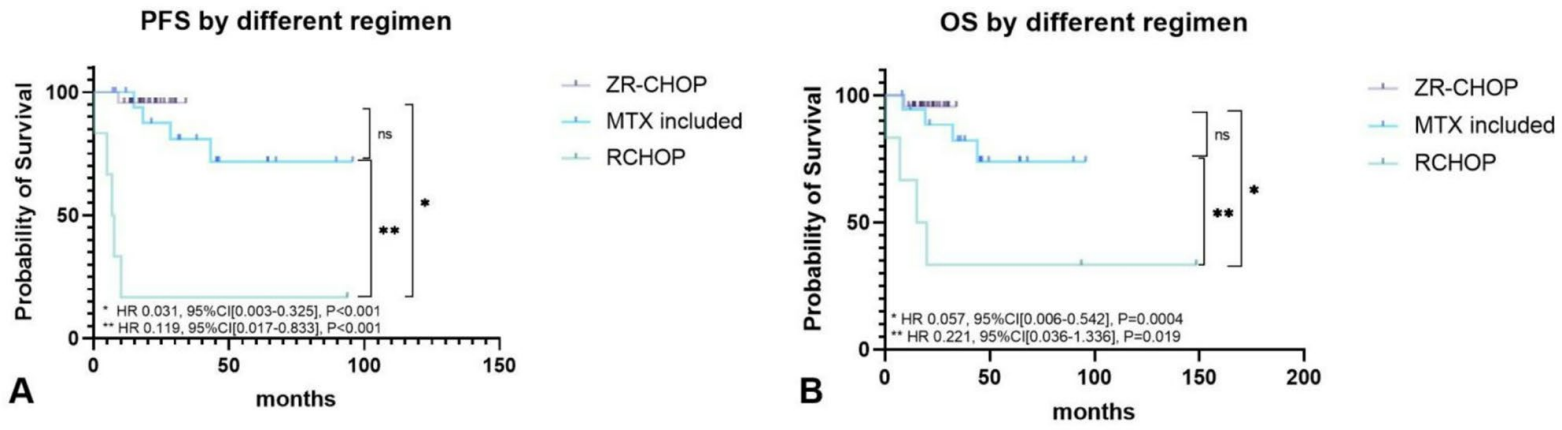
Survival of patients by different treatment. **A**/**B**. PFS and OS of patients treated by different therapy

**Fig. 3:**
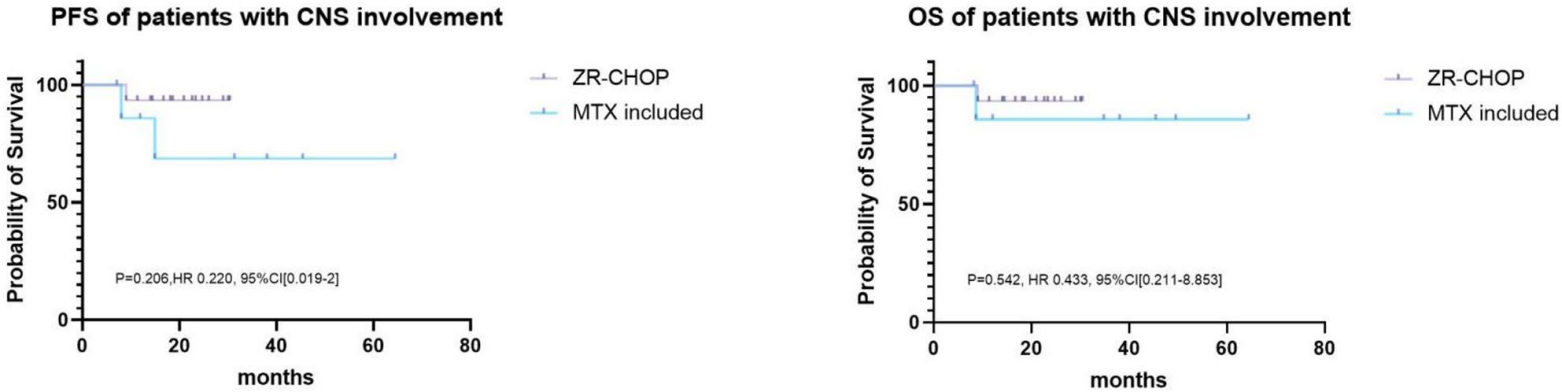
Survival of patients with CNS involvement receiving different treatment

Considering the effect of treatment on survival, we studied response rate, PFS and OS of patients treated by different regimens. We firstly compared baseline features of patients receiving different treatment and found that there was only significant difference in International Prognostic Index(IPI, *P* = 0.022). All(6/6) the patients receiving palliative care and 86.4%(19/22) of patients treated by ZR-CHOP were in IPI high risk group, while the fraction in the other two therapies was 50% (10/20, MTX included therapy) and 66.7%(4/6, RCHOP), respectively (Table 3). Both the ZR-CHOP(*n* = 22) and MTX-based therapy (*n* = 20) therapies induced higher CRR compared to R-CHOP(*n* = 6). Specifically, the CR rate was 90.9% (20/22) in the ZR-CHOP group, 90.0% (18/20) in the MTX-based therapy group, and 66.7% (4/6) in the R-CHOP group. Although the differences did not reach statistical significance (*P* = 0.309, Fisher’s exact test), likely due to the small sample size, ZR-CHOP demonstrated a numerically superior response. This profound initial efficacy laid the foundation for the superior long-term survival outcomes observed with these regimens.

In progression-free survival (PFS) analysis, both ZR-CHOP (*n* = 22; 1 event) and MTX-based therapy (*n* = 20; 4 events) significantly outperformed R-CHOP (*n* = 6; 5 events), with hazard ratios of 0.031 (95% CI, 0.003–0.325; *P* < 0.0001) and 0.119 (95% CI, 0.017–0.833; *P* < 0.0001), respectively. The PFS benefit of ZR-CHOP was numerically greater than that of MTX-based therapy, but the difference between them was not statistically significant (HR, 0.448; 95% CI, 0.072–2.781; *P* = 0.411). A consistent advantage was observed in overall survival (OS). Both ZR-CHOP (1 death) and MTX-based therapy (4 deaths) demonstrated superior OS compared to R-CHOP (4 deaths), with HRs of 0.057 (95% CI, 0.006–0.542; *P* = 0.0004) and 0.221 (95% CI, 0.036–1.336; *P* = 0.019), respectively. Similarly, no significant OS difference was detected between the two experimental regimens (HR, 0.516; 95% CI, 0.080–3.349; *P* = 0.480).Palliative care yielded a dismal prognosis, median PFS and OS were both 0.9 months.(Fig. 2A/B).

Given the difference in median follow-up between the ZR-CHOP (20.1 months) and MTX-based therapy (38.0 months) groups, we compared landmark PFS rates at time points for which robust data were available for both cohorts. The 12-month PFS rate was 95.7% for ZR-CHOP(*n* = 22) compared to 100% for MTX-based therapy(*n* = 20). The 18-month PFS rate was 95.7% versus 87.5%, respectively. This analysis within the first 18 months—a critical period for relapse in aggressive lymphomas—demonstrates the potent and early efficacy of the ZR-CHOP regimen. Subgroup analysis of patients with CNS involvement—a population with traditionally poor prognosis—revealed that ZR-CHOP yielded excellent and comparable outcomes to MTX-based therapy. Despite the high-risk profile of this cohort (63.6% in the ZR-CHOP group and 40.0% in the MTX-based group, Table 2), both regimens achieved remarkable disease control, with neither median PFS nor OS being reached in either group. The hazard ratios for progression and survival showed no evidence of inferiority for ZR-CHOP (PFS: HR 0.22, 95% CI 0.02-2.00, *P* = 0.206; OS: HR 0.43, 95% CI 0.21–8.85, *P* = 0.542), although the wide confidence intervals, likely due to the limited number of events in this subgroup, preclude definitive conclusions regarding superiority(Fig. 3).

In the whole cohort, there were two patients experiencing CNS relapse, one was treated by RCHOP, the other had been given intravenous MTX (1 g/m^2^) as CNS prophylaxis. In both cases, the relapse occurred within one year (5.0 months for the patient treated by RCHOP and 10.1 months for MTX included therapy).

The ZR-CHOP regimen demonstrated a manageable safety profile consistent with the R-CHOP backbone. The most common grade 3/4 adverse events were hematological. Non-hematological toxicities included infections (54.5%), with fungal infections occurring in 9.1% of patients, all of which resolved with appropriate management. A systematic assessment of key BTK inhibitor class effects revealed no grade ≥ 3 bleeding events, no episodes of atrial fibrillation, and no new-onset hypertension attributed to zanubrutinib(Table S2). No adverse events led to permanent treatment discontinuation.

### Prognostic indicators for clinical outcome (Table S3)

To mitigate potential confounding by calendar era and indication, we performed a multivariable Cox regression analysis adjusting for baseline clinical features such as age, performance status, IPI risk group, extranodal involved site, CNS involvement, and treatment. Since there was no difference of survival between ZR-CHOP and MTX containing therapy, we defined them as CNS oriented therapy, comparing to non-CNS oriented therapy (including RCHOP and palliative care). Univariate analysis showed that CNS oriented therapy was the only predictor for better PFS(HR 95%CI 0.063[0.022–0.179], *P* < 0.001) and OS(HR 95%CI 0.093[0.031–0.280], *P* < 0.001). Age, gender, IPI, ECOG, LDH, anemia, thrombocytopenia, hypoalbuminemia, presenting with shock, hypoxia, or HLH, and involvement of lung, nervous system, or skin, cell of origin, MCD subtype did not have significant impact on the survival outcomes. Bone marrow involvement seemed to have a poorer OS(*P* = 0.062). Patients who experienced progression of disease(400.3pg/ml) had a similar mean level of IL-10 comparing to those who were progression free(514.5pg/ml)(*P* = 0.497, Mann-Whitney U test). This was the same for patients who died(439.5pg/ml) versus those were alive(501.1pg/ml)(*P* = 0.728). We also compared the plasma ctDNA concentration of patients who had disease progression(120.72ng/ml) to those who were disease free(151.44ng/ml) but found no differences(*P* = 0.75). This was also the truth for patients who died comparing to those who were alive(120.72ng/ml vs.151.44ng/ml, *P* = 0.75). When performing multivariate analysis, we included parameters which had a p value < 0.1 in univariate analysis and found that CNS oriented therapy was the only factor that predicted better outcome (Table S3).

## Discussion

Our 13-year single-center experience demonstrates significant advancements in IVLBCL management through diagnostic innovation and molecularly-guided therapy. The The development and implementation of our IL-10/RSB/ctDNA diagnostic algorithm has successfully addressed the longstanding challenge of PET/CT-negative cases, which represented 22.9% of our cohort. Our molecular profiling confirmed the predominance of the MCD subtype (*MYD88/CD79B*-mutated) in IVLBCL, with *MYD88L265P* variants in 65%(11/17) of cases and *CD79B* variants in 41%(7/17) of cases. This molecular landscape provided the rationale for zanubrutinib use, as ZR-CHOP yielded 100% CNS control despite 63.6% baseline involvement and comparable efficacy to MTX-based therapy. Given the ultra-orphan nature of IVLBCL and the retrospective, real-world design of this study spanning 13 years, a prospective power analysis for sample size calculation was not feasible. The sample size (*n* = 54) represents one of the largest single-center cohorts for this disease to date. The statistical analyses performed are therefore descriptive and exploratory in nature, with the dramatic effect sizes observed (e.g., HR for PFS of 0.031 for ZR-CHOP vs. R-CHOP) providing strong clinical, if not solely statistical, evidence of efficacy.

As a rare entity of DLBCL, IVLBCL always posed a diagnostic challenge because of nonspecific presenting symptom and lack of a lymph node or mass for biopsy [3]. For some patients, diagnosis was only made when the general condition deteriorated, and patients missed the best opportunity for treatment. In recent years, random skin biopsy (RSB) of uninvolved skin became a reputable tool, with a sensitivity of 77.8% and specificity 98.7% for diagnosis of IVLBCL. The positive and negative predictive values of RSB were 96.6% and 90.6%, respectively [6]. Investigators further developed an IVLBCL-probability scoring system to guide the application of RSB. This score comprised the following 4 components: general symptom, organ specific symptom, soluble interleukin-2 receptor (sIL-2R) level and LDH. They suggested that the application of RSB could be limited to the high-probability group [15]. However, sIL-2R was indeed not a specific biomarker for IVLBCL. It would be elevated in many other types of non-Hodgkin lymphoma (NHL) [16–18]. In our previous study, the serum levels of IL-10 in IVLBCL were significantly higher than those in control groups with DLBCL, not otherwise specified (DLBCL-NOS) included (*P* < 0.0001), with a median level of 490 pg/mL (range, 5-1000) versus 5 pg/mL (range, 5-75.3). It had a diagnostic sensitivity of 80% and specificity of 100% for IVLBCL when a cut-off value of 95.65 pg/mL was set [13]. Our IL-10/RSB/ctDNA diagnostic approach proved particularly valuable for diagnostically challenging patients presenting with non-specific symptoms (90.7% B symptoms), poor performance status (59.3% ECOG 3–4) and multiorgan involvement (median 3 sites). The impact of this diagnostic evolution on case ascertainment is summarized in Supplementary Figure S1A, with quantitative data presented in Table 2.

Our study must be interpreted in the context of evolving diagnostic practices. The rising incidence of PET/CT-negative cases over the study period is a direct consequence of the implementation of our IL-10/RSB algorithm, not a change in disease epidemiology. The fact that no such cases were diagnosed in the 2010–2016 era is a testament to the historical under-ascertainment of this specific patient phenotype. Therefore, cross-era comparisons of outcome should not be viewed as a simple comparison of homogeneous groups, but rather as evidence of how diagnostic evolution has progressively allowed us to identify and treat a broader spectrum of IVLBCL patients, ultimately improving overall disease management. Meanwhile, A nuanced analysis of the time from symptom onset to diagnosis reveals a critical insight: the interval was not shortened but was, in fact, numerically longer in the era of systematic IL-10/RSB testing. This phenomenon underscores the fundamental purpose of the IL-10/RSB approach: it was not designed to marginally accelerate diagnosis in straightforward cases, but to definitively establish a diagnosis in otherwise unsolvable ones. The method successfully captured a subset of patients who had undergone prolonged, often fruitless, diagnostic odysseys elsewhere. Therefore, the slightly longer average diagnosis time powerfully confirms that our approach succeeded in its primary goal: identifying and rescuing the very patients who would have otherwise remained undiagnosed.This observation, which initially appears counterintuitive, powerfully refutes the concern of lead-time bias. We interpret this as a manifestation of referral and selection bias. The IL-10/RSB algorithm served as a powerful tool for resolving diagnostically challenging cases that had exhausted conventional evaluations at other institutions. Consequently, our center increasingly became a referral destination for patients with prolonged, undiagnosed illnesses. The incorporation of these “diagnostic orphans”—patients with extensive pre-referral evaluation periods—into our cohort naturally extended the average diagnostic interval. Thus, the longer time to diagnosis does not reflect a failure of the method but rather stands as testament to its unique ability to provide definitive diagnoses for the most elusive cases.

Besides improved diagnostic methods, we also understanded this entity better in perspective of genomic profile recent years. In the year of 2018, Schrader and colleagues first reported a high prevalence of *MYD88 L265P* mutation (44%) and *CD79B Y196* mutation (26%) in 25 IVLBCL patients [19]. In 2021, Shimada et al. performed whole-exome sequencing (WES) of 21 IVLBCL patients, 18 of whom were used plasma-derived cell-free DNA (cfDNA). Results showed conspicuously higher frequencies (compared with nodal DLBCL) of mutation in *MYD88*(57%), *CD79B* (67%), *SETD1B* (57%), and *HLA-B* (57%) [20]. These conclusions were confirmed by another study performing next-generation sequencing analysis of 15 cases of IVLBCL, which also showed the relevance of mutations in B-cell receptor/nuclear factor-κB signaling and immune escape pathways. In their study, the most frequently mutated gene was *PIM1* (9/15, 60%), followed by *MYD88L265P* and *CD79B* (8/15, 53% each) [21]. Our study has similar results, *PIM1*,* MYD88L265P* and *CD79b* variants was found in 71%(12/17), 65%(11/17) and 41%(7/17) of 17 patients, respectively. The high prevalence of *MYD88 L265P* in our cohort is of pathogenic significance, as this variant constitutively activates the NF-κB signaling pathway and promotes cell survival, a hallmark of the MCD subtype of DLBCL. Concurrent *CD79B* mutations, frequently affecting the immunoreceptor tyrosine-based activation motif (ITAM), work synergistically with *MYD88 L265P* by amplifying B-cell receptor signaling and preventing antigen-induced cell death, further driving lymphomagenesis in IVLBCL.Our ctDNA analysis, while limited to a subset of patients (*n* = 17), revealed a characteristic mutational profile that is biologically and clinically informative. However, the reported variant frequencies are descriptive of this molecularly characterized subgroup and not necessarily representative of the entire IVLBCL population. This insight was, however, critical for motivating the evaluation of targeted therapies like zanubrutinib in our center.

Our therapeutic approach to IVLBCL has evolved significantly through three distinct phases. Initially, patients received standard R-CHOP therapy, mirroring treatment for conventional DLBCL. Recognizing the high risk of CNS involvement, we subsequently incorporated methotrexate-based CNS prophylaxis (1 g/m² for high-risk patients) and high-dose MTX (3.5 g/m²) for those with established CNS disease, building on our experience with other high-risk lymphomas [14]. This CNS-directed strategy yielded outcomes comparable to the PRIMEUR-IVL study, which demonstrated 76% 2-year PFS and only 3% CNS relapse at 2 years with intensive MTX-based regimens [12]. The current therapeutic era has been shaped by molecular insights into IVLBCL pathogenesis. The recognition that most cases belong to the MCD subtype (characterized by MYD88/CD79B mutations) [22], combined with promising results from the Phoenix study showing 100% 3-year EFS with ibrutinib in MCD-type DLBCL, prompted our evaluation of zanubrutinib-based therapy. As a next-generation BTK inhibitor with demonstrated CNS penetration [23], zanubrutinib (BRUKINSA™) was incorporated into our ZR-CHOP regimen. This approach has shown comparable efficacy to MTX-based therapy while offering particular advantages in CNS disease control, building on prior observations of its 81.8% response rate in CNS-involved aggressive B-cell lymphomas [24]. To our knowledge, this represents the first systematic application of targeted therapy in this rare disease entity.

We must address the role of zanubrutinib maintenance in the ZR-CHOP regimen. It is plausible that the two-year maintenance phase contributed to the sustained remission, particularly in preventing late relapses. However, several lines of evidence suggest that the profound efficacy is primarily driven by the initial ZR-CHOP induction therapy. The remarkably high complete remission rate achieved after induction, coupled with the superior PFS rates observed within the first 18 months—a period largely reflective of induction efficacy—strongly supports this conclusion. While maintenance may function to consolidate these deep responses, it is unlikely to be the principal factor responsible for the dramatic superiority over R-CHOP. This insight is encouraging, as it suggests that the core ZR-CHOP induction backbone is already highly effective in controlling this aggressive disease.

In conclusion, Over our 13-year experience, we have established an optimized management paradigm for IVLBCL that integrates diagnostic and therapeutic advances. The combination of serum IL-10 testing with random skin biopsy significantly improves early detection, particularly for PET/CT-negative cases, while ctDNA analysis confirms the predominance of the MCD subtype in most patients. Our pioneering use of zanubrutinib in the ZR-CHOP regimen demonstrates promising efficacy, especially for CNS involvement, offering a more accessible targeted therapy option in resource-limited settings. These advances address critical unmet needs in IVLBCL management, though further validation through multicenter prospective studies remains warranted.

## Supplementary Information

Below is the link to the electronic supplementary material.

Supplementary Material 1

Supplementary Material 2

## Data Availability

The de-identified datasets generated and analyzed during the current study are not publicly available due to patient privacy concerns but are available from the corresponding author (Y.Z.) on reasonable request. Data requests will be reviewed and approved by the institutional ethics committee to ensure compliance with ethical obligations.
