# Supplementary material for: Breaking diagnostic and therapeutic barriers in intravascular large B-cell lymphoma: A 13-year real-world study from China
Source: Orphanet J Rare Dis. 2026 Feb 11;21:98. doi: 10.1186/s13023-026-04226-4 (PMC12997734; doi:10.1186/s13023-026-04226-4)

**Table S1 Features and diagnosis of patients with negative PET/CT**

| **Gender** | **Age** | **Ann Arobor  stage** | **ECOG** | **LDH** | **IPI** | **Symptoms** | **sIL-10 pg/ml** | **Presence of skin rash** | **RSB applied** | **Diagnostic biopsy site** |
| --- | --- | --- | --- | --- | --- | --- | --- | --- | --- | --- |
| M | 60 | IVB | 3 | 604 | 4 | peripheral edema, hypotension | NA | yes | yes | skin |
| M | 52 | IVA | 3 | 317 | 3 | right lower limb weakness | 1000 | no | yes | skin |
| F | 62 | IVB | 4 | 2035 | 3 | fever, hypotension | 183.0 | no | yes | lymph node |
| F | 57 | IVB | 3 | 1111 | 4 | fatigue, peripheral edema, acute renal insufficiency | 1000.0 | no | yes | skin |
| M | 58 | IVB | 4 | 559 | 4 | fever, peripheral edema, abdomincal distension | 331.0 | yes | yes | skin, bone marrow |
| M | 59 | IVB | 4 | 1370 | 4 | fever, dyspnea | 1000.0 | no | yes | skin |
| M | 44 | IVB | 3 | 194 | 4 | fever, dyspnea, hypotension | 293.0 | no | yes | skin |
| F | 51 | IVB | 1 | 204 | 3 | fever, cough, dyspnea | 153.7 | no | yes | lung |
| M | 48 | IVB | 4 | 723 | 5 | fever | 210 | no | yes | spleen |
| M | 73 | IVB | 4 | 431 | 5 | fever, short of breath | 11.6 | no | yes | skin |
| M | 57 | IVB | 1 | 447 | 4 | fever, peripheral edema | 1000 | no | yes | skin |

**Abrrevations:** ECOG: Eastern Cooperative Oncology Group, LDH: lactate dehydrogenase, IPI: international prognostic index, sIL-10: serum interleukin-10, RSB: random skin biopsy.

**Table S2. Safety profile of ZR-CHOP regimen**

|  | Any grade | Grade3 | Grade4 |
| --- | --- | --- | --- |
| **Hematological toxicities（per cycle，N=143）** | | | |
| Neutropemia | 64（44.8%） | 14（9.8%） | 26（18.2%） |
| Anemia | 20（14.0%） | 2（1.4%） | 0（0%） |
| Thrombocytopenia | 7（4.9%） | 2（1.4%） | 1（0.7%） |
| **Non-hematological toxicities（per patient，N=22）** | | | |
| Nausea | 16（72.7%） | 1（4.5%） | 0（0%） |
| Infection | 12（54.5%） | 12（54.5%） | 0（0%） |
| Fungi infection | 2（9.1%） | 2（9.1%） |  |
| Fatigue | 7（31.8%） | 0（0%） | 0（0%） |
| Infusion related reaction | 6（27.3%） | 2（9.1%） | 2（9.1%） |
| Febrile neutropenia | 6（27.3%） | 5（22.7%） | 1（0.7%） |
| Peripheral neuropathy | 4（18.2%） | 0（0%） | 0（0%） |
| Aspartate/Alanine aminotransferase increased | 3（13.6%） | 0（0%） | 0（0%） |
| Tumor lysis syndrome | 1（4.5%） | 0（0%） | 0（0%） |
| Bleeding | 2（9.1%） | 0（0%） | 0（0%） |
| Atrial fibrillation | 0（0%） | 0（0%） | 0（0%） |

**Table S3 Univariate and multivariate analysis for risk factors**

| **Characteristics** | **Univariate analysis** | | | | **Multivariate analysis** | | | |
| --- | --- | --- | --- | --- | --- | --- | --- | --- |
|  | **PFS** | | **OS** | | **PFS** | | **OS** | |
|  | **HR 95%CI** | **P** | **HR 95%CI** | **P** | **HR 95%CI** | **P** | **HR 95%CI** | **P** |
| Age >60 vs. ≤60 | 0.739(0.276-1.976) | 0.547 | 0.738(0.259-2.110) | 0.571 |  |  |  |  |
| Sex male vs. female | 0.799(0.296-2.158) | 0.659 | 1.126(0.389-3.258) | 0.826 |  |  |  |  |
| ECOG 3-4 vs. 0-2 | 1.383(0.491-3.895) | 0.540 | 2.039(0.677-6.145) | 0.206 |  |  |  |  |
| IPI 4-5 vs. 0-3 | 1.488(0.477-4.646) | 0.494 | 1.908(0.527-6.907) | 0.325 |  |  |  |  |
| Presenting with vs. without |  |  |  |  |  |  |  |  |
| shock | 0.706(0.159-3.137) | 0.648 | 0.902(0.200-4.069) | 0.893 |  |  |  |  |
| hypoxia | 1.988(0.721-5.483) | 0.184 | 2.096(0.701-6.272) | 0.186 |  |  |  |  |
| HLH | 0.452(0.129-1.590) | 0.216 | 0.551(0.153-1.982) | 0.362 |  |  |  |  |
| Involved site |  |  |  |  |  |  |  |  |
| lung | 1.227(0.444-3.392) | 0.693 | 1.348(0.449-4.045) | 0.595 |  |  |  |  |
| CNS | 1.182(0.436-3.206) | 0.742 | 1.327(0.456-3.864) | 0.604 |  |  |  |  |
| skin | 0.441(0.142-1.374) | 0.158 | 0.579(0.180-1.862) | 0.359 |  |  |  |  |
| **bone marrow** | 2.072(0.765-5.614) | 0.152 | 2.847(0.95-8.531) | **0.062** | 1.469(0.449-4.806) | 0.524 | 0.630(0.200-1.988) | 0.431 |
| Anemia | 0.997(0.346-2.874) | 0.996 | 1.818(0.506-6.528) | 0.360 |  |  |  |  |
| Thrombocytopenia | 0.639(0.237-1.724) | 0.377 | 0.855(0.299-2.442) | 0.769 |  |  |  |  |
| Hypoalbuminemia | 1.705(0.344-3.357) | 0.901 | 2.399(0.535-10.749) | 0.253 |  |  |  |  |
| **LDH elevated** | 1.355(0.177-10.393) | 0.770 | 1.033(0.133-8.057) | 0.975 |  |  |  |  |
| LDH>1000U/L | 0.756(0.274-2.087) | 0.589 | 0.677(0.227-2.022) | 0.485 |  |  |  |  |
| GCB vs. nonGCB | 0.317(0.050-2.015) | 0.223 | 0.317(0.045-2.222) | 0.247 |  |  |  |  |
| MCD subtype vs. not MCD | 0.289(0.009-9.465) | 0.488 | 0.289(0.009-9.465) | 0.488 |  |  |  |  |
| **CNS oriented therapy** | 0.063(0.022-0.179) | **<0.001** | 0.093(0.031-0.280) | **<0.001** | 0.051(0.015-0.128) | **<0.001** | 0.107(0.034-0.339) | **<0.001** |

**Abrrevations**: PFS, progression free survival, OS: overall survival, ECOG: Eastern Cooperative Oncology Group, IPI: international prognostic index, HLH: hemophagocytic lymphohistiocytosis, CNS: central nervous system, LDH: lactate dehydrogenase, GCB: germinal center B cell-like, MCD (based on the co-occurrence of **M**YD88L265P and **CD**79B mutations), CNS: central nervous system.

**Figure S1 A. Patient Flow Diagram: Diagnostic Strategy Evolution and Cohort Formation over the 13-Year Study Period**


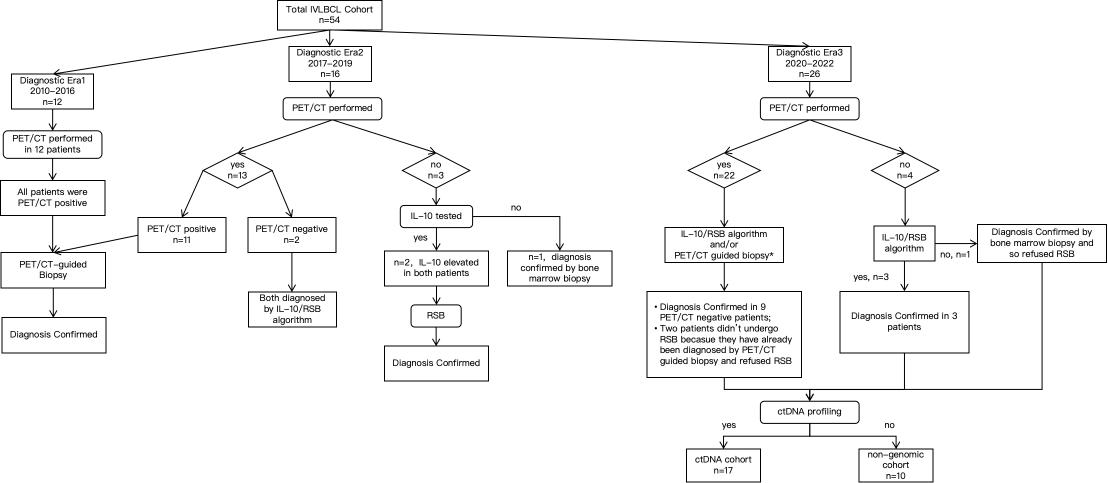


Note：All patients who did not undergo PET/CT were too critically ill to tolerate transport for the procedure.

* In this phase(2020-2022), serum IL-10 was measured in all patients, and the majority subsequently underwent routine random skin biopsy (RSB)

**Figure S1 B. Pathological biopsy site.**


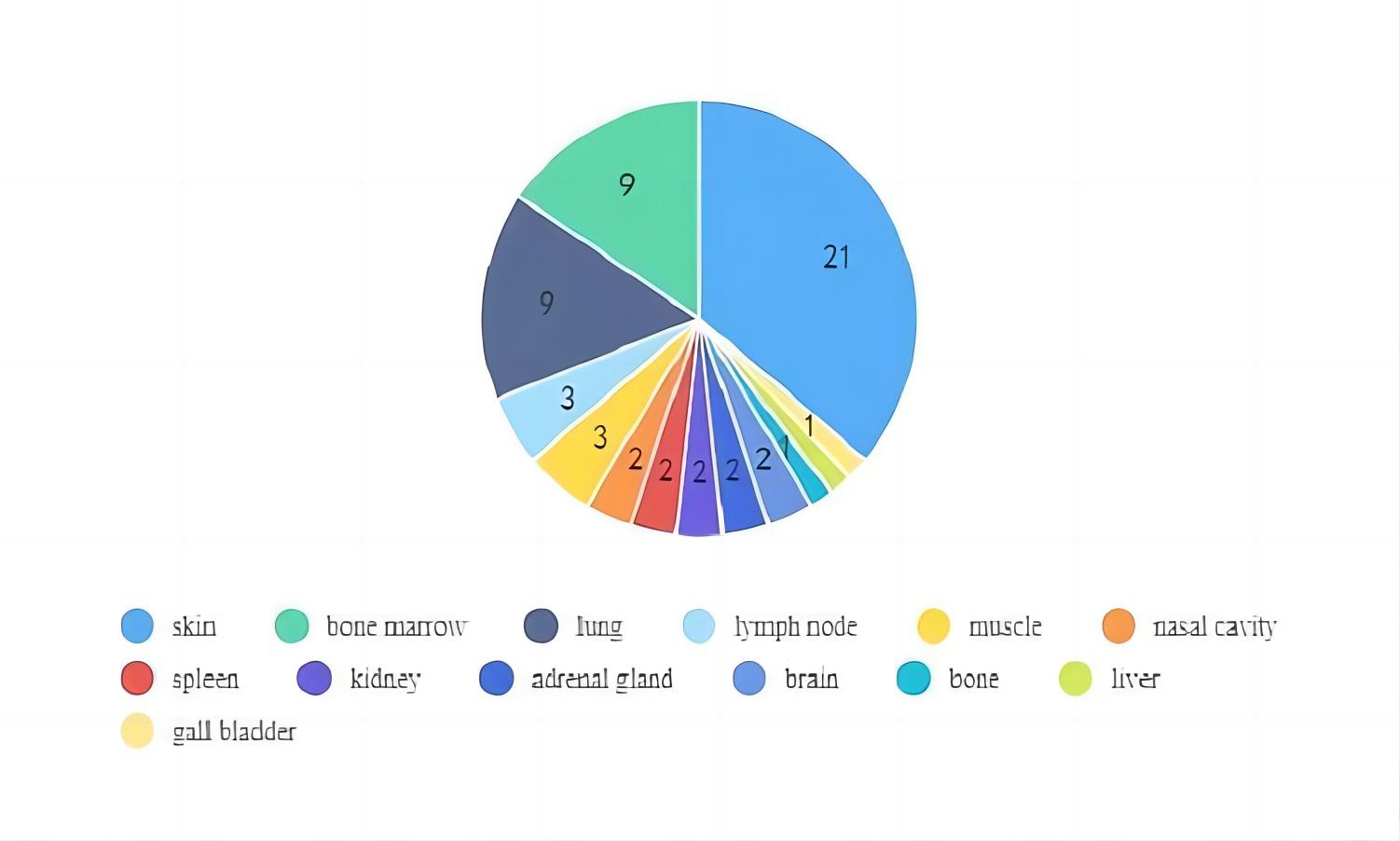


**Figure S2. Genetic alteration landscape of 17 IVLBCL patients**

Mutations, CNAs and SVs detected in 17 patients with IVLBCL are shown. The vertical line indicates 1 patient. Percentage on the left indicated the fraction of patients with altered genes labeled on the right. The column chart on top showed the composition of alteration of each patient. The column chart on the right revealed the types of alteration of each gene. Genes were displayed according to pathways they belonged to.


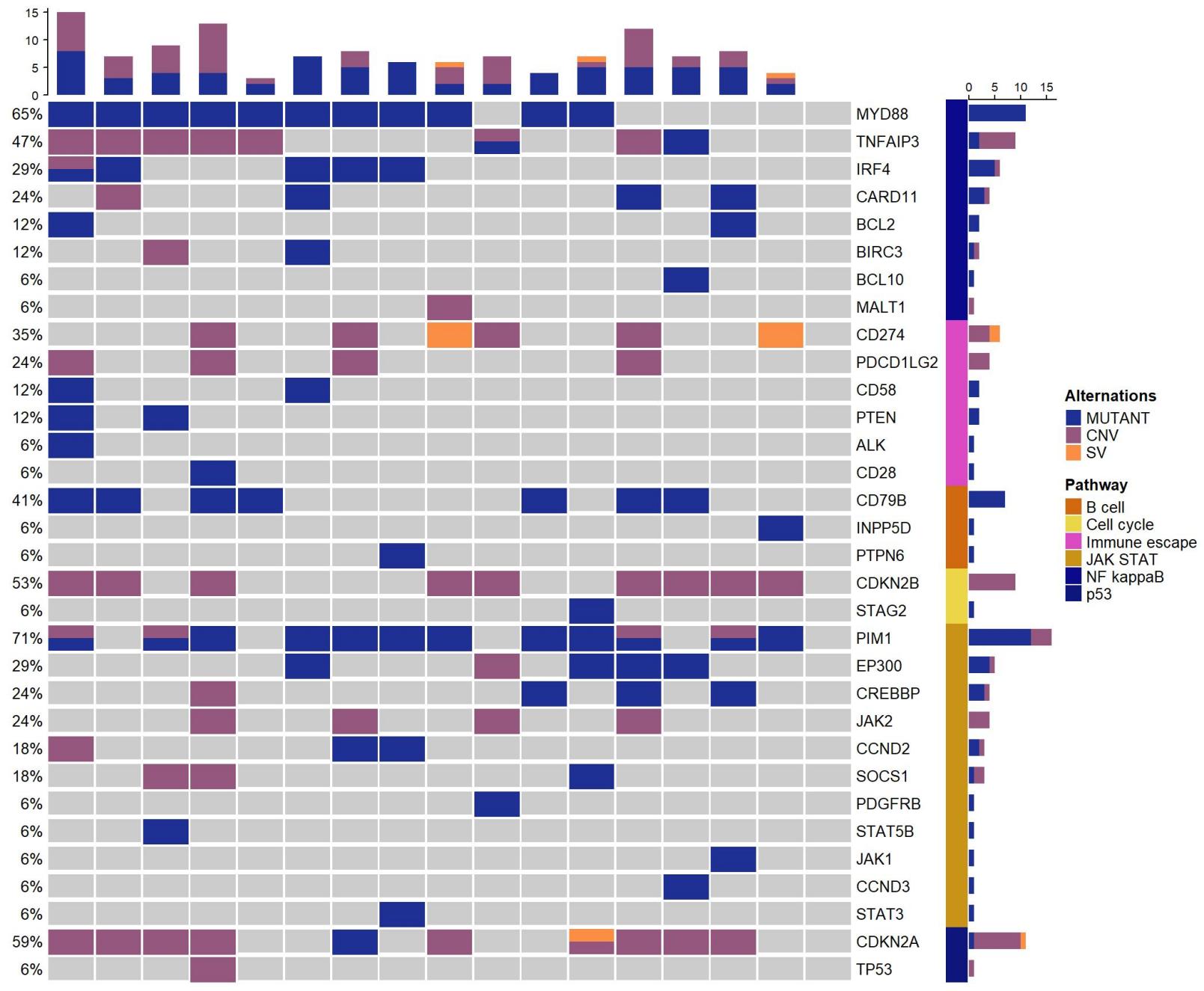

Supplement: Supplementary file 1 — Supplementary Material 1 [file 13023_2026_4226_MOESM1_ESM.docx]
